# *γ*-MYN: a new algorithm for estimating Ka and Ks with consideration of variable substitution rates

**DOI:** 10.1186/1745-6150-4-20

**Published:** 2009-06-16

**Authors:** Da-Peng Wang, Hao-Lei Wan, Song Zhang, Jun Yu

**Affiliations:** 1 CAS Key Laboratory of Genome Sciences and Information, Beijing Institute of Genomics, Chinese Academy of Sciences, Beijing 100029, PR China; 2 Graduate University of Chinese Academy of Sciences, Beijing 100039, PR China; 3 Institute of Computing Technology, Chinese Academy of Sciences, Beijing 100080, PR China

## Abstract

**Background:**

Over the past two decades, there have been several approximate methods that adopt different mutation models and used for estimating nonsynonymous and synonymous substitution rates (Ka and Ks) based on protein-coding sequences across species or even different evolutionary lineages. Among them, MYN method (a Modified version of Yang-Nielsen method) considers three major dynamic features of evolving DNA sequences–bias in transition/transversion rate, nucleotide frequency, and unequal transitional substitution but leaves out another important feature: unequal substitution rates among different sites or nucleotide positions.

**Results:**

We incorporated a new feature for analyzing evolving DNA sequences–unequal substitution rates among different sites–into MYN method, and proposed a modified version, namely *γ *(gamma)-MYN, based on an assumption that the evolutionary rate at each site follows a mode of *γ*-distribution. We applied *γ*-MYN to analyze the key estimator of selective pressure ω (Ka/Ks) and other relevant parameters in comparison to two other related methods, YN and MYN, and found that neglecting the variation of substitution rates among different sites may lead to biased estimations of ω. Our new method appears to have minimal deviations when relevant parameters vary within normal ranges defined by empirical data.

**Conclusion:**

Our results indicate that unequal substitution rates among different sites have variable influences on ω under different evolutionary rates while both transition/transversion rate ratio and unequal nucleotide frequencies affect Ka and Ks thus selective pressure ω.

**Reviewers:**

This paper was reviewed by Kateryna Makova, David A. Liberles (nominated by David H Ardell), Zhaolei Zhang (nominated by Mark Gerstein), and Shamil Sunyaev.

## Background

Comparative sequence analysis is a powerful tool for biologists to study evolutionary relationship among animals and plants across diverse taxonomic lineages [1-3]. Pair-wise sequence comparison is perhaps the simplest comparative analysis for phylogeny for two reasons [4]. First, calculating pair-wise distances is the initial step for distance-matrix methods of phylogeny reconstruction. Second, Markov-process models of nucleotide substitution used in distance calculations lay a foundation for likelihood and Bayesian analyses. One of the sophisticated methods is to estimate nonsynonymous and synonymous substitution rates for interrogating sequence dynamics and constructing phylogenetic trees. Since Ka and Ks represent the number of substitutions per nonsynonymous and synonymous site, respectively, these parameters (or often their ratio Ka/Ks or ω) are important for the estimation of evolutionary rates. The indications of Ka < Ks (ω < 1), Ka > Ks (ω > 1), and Ka = Ks (ω = 1) on evolutionary trends are negative (purifying), positive (adaptive), and neutral mutations, respectively. Ka and Ks can be estimated based on approximate methods, which typically involve three essential steps [4]: (1) counting the number of synonymous (S) and nonsynonymous (N) sites among targeted sequences, (2) counting the number of synonymous (S_d_) and nonsynonymous (N_d_) substitutions between two orthologous sequences, and (3) calculating the number of synonymous (d_s_) and nonsynonymous (d_n_) substitutions per site after correcting for multiple substitutions. Most of the methods assume simplified nucleotide substitution paths and involve *ad hoc *data treatments that are not well-justified [5,6]. For instance, NG (Nei-Gojobori) method, a commonly-used approximate method in the early days, considers all possible evolutionary courses among compared DNA sequences and assumes that each nucleotide can be substituted with any of three other nucleotides at equal rate when it counts both sites and substitutions [7]. It adopts Jukes-Cantor's one parameter formula only to correct for multiple substitutions. Another example, LWL (Li-Wu-Luo) method, classifies sites and substitutions as *i*-fold degenerate sites (*i *= 0, 2, 4) and considers unequal rates between transitional and transversional changes only when it counts substitutions, but considers equal rates when counting sites [8]. A modified LWL, LPB (Li-Pamilo-Bianchi) method corrects for bias in counting sites by using different formulas for Ka and Ks estimation, which differentiate LPB from LWL method [9,10]. Versions of LWL and LPB methods were also proposed by distinguishing two-fold degenerate sites and substitutions, taking the account of the transition/transversion rate bias when counting sites and correcting for arginine codons [11,12].

Among approximate methods, YN (Yang-Neilsen) method made significant progress through consideration of transition/transversion rate and nucleotide frequency biases [13]. Based on YN method, we recently proposed a modified YN method (MYN) to distinguish substitutions between purines (A/G) and between pyrimidines (T/C) [13,14]. MYN incorporates most of the major features of sequence evolution but assumes that different sites in sequences evolve the same way and at the same rate. This assumption is somewhat less thorough, and accumulating evidence of rate variation over sites is rather overwhelming [15-20]. Since mutation rates certainly vary among sites, and mutations at different sites may be fixed or drifting at different rates due to their versatile roles in the structure and function of gene products (mostly proteins albeit RNAs also fold into different conformations), unequal nucleotide frequencies, different codon usage among species, and variation of substitution rates among different sites should all be taken into account, allowing for significant yet maybe incremental improvements on various parameter estimations. Some sixteen years ago, one of the pioneers of this field, Ziheng Yang suggested *γ*-distribution (gamma-distribution) as an adequate approximation based on his intensive comparative analysis on several continuous distributions leveraging on sequence data from the globin genes [21]. As *γ*-distribution has been frequently used in estimating sequence divergence [7,22-27], we adopt it to formulate an improved approximate method, denoted as *γ*-MYN, based on MYN method [14]. In this method, we assume that nucleotide substitutions follow *γ*-distribution because negative binomial distribution is known to be generated when Poisson parameter *γ *varies according to a particular *γ *distribution among sites [28]. We would like to emphasize that the *γ *distribution here refers to raw mutation rate rather than *γ *distribution of ω itself. It has been proposed that nucleotide substitution in coding region is context-dependent [29], and therefore, substitution rates depend on not only the neighboring sequences but also their functional constraints and models that allow for the correlation of substitution rates at adjacent sites were also developed [30,31]. However, as these models tend to produce results similar to the simple gamma model and variations of α can make the distribution suitable for accommodating different levels of rate variations in various datasets [31], we chose the simple gamma distribution as the depiction of raw various mutation rates. Since YN and MYN methods perform better as compared to numerous other methods [12] and MYN improves the performance of YN for most parameter combinations [14], we focus on evaluating the performance of *γ*-MYN by comparing it to YN and MYN under variable conditions. The definitions of symbols used in Ka and Ks estimations are listed in Table 1.

**Table 1:** Symbols used in Ka and Ks calculation

**Symbol**	**Definition**
S	Number of synonymous sites
N	Number of nonsynonymous sites
Ks	Synonymous substitution rate
Ka	Nonsynonymous substitution rate
ω	Estimator of selective strength, ω = Ka/Ks
S_d_	Number of synonymous substitutions
N_d_	Number of nonsynonymous substitutions
*t*	Divergence time between two sequences, the expected number of nucleotide substitutions per codon, *t *= (Ks × 3S + Ka × 3N)/(S + N)
α	The parameter of gamma distribution
κ	Ratio of transitional rate/transversional rate
κ_R_	Ratio of transitional rate between purines to transversional rate, κ_R _= α_R_/β
κ_Y_	Ratio of transitional rate between pyrimidines to transversional rate, κ_Y _= α_Y_/β
g_N_	Frequency of nucleotide N, N ∈ [T, C, A, G]
α_R_	Transitional rate between purines
α_Y_	Transitional rate between pyrimidines
β	Transversional rate

## Results

### Computer simulation

Computer simulation is a routine approach for evaluating computational procedures of different algorithms. In molecular phylogeny, one major approach for simulating DNA sequence evolution is to generate an ancestral sequence for the root of a tree and "evolve" it along the tree building process according to substitution models, branch lengths, and substitution parameters [11,32-35]. This approach can be implemented in the evolver program in the PAML (Phylogenetic Analysis by Maximum Likelihood [36]) package, which usually uses nucleotide or amino acid sequence data to simulate evolving protein-coding sequences. To assess the advantages of γ-MYN in comparison with YN and MYN, we generated three groups of simulated sequences with the PAML package: (1) equal codon frequencies, (2) human frequencies (based on human protein-coding genes from the ENSEMBL database) [37] and (3) rice frequencies (based on rice protein-coding genes) [38]. We also generated 2,000 sequence pairs with 1,200 bp in length for examining the effect of different parameters.

### Consistency analysis and effect of codon frequencies

**Figure 1:**
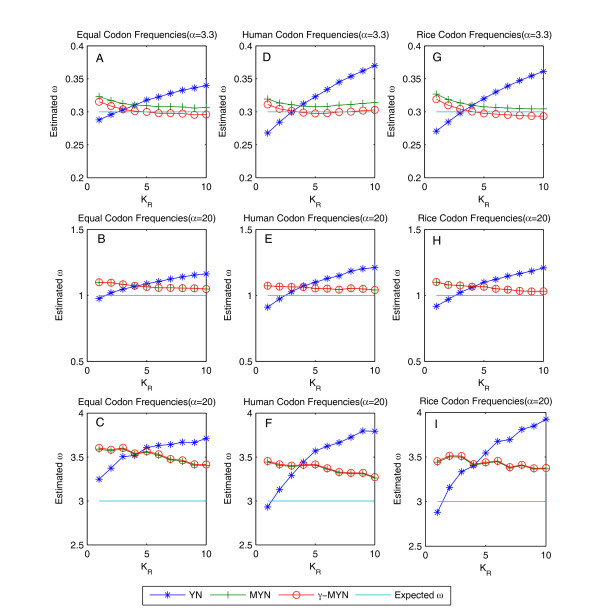
**Estimated ω based on YN, MYN, and *γ*-MYN**. We plotted ω values estimated by YN, MYN, and *γ*-MYN when κ_Y _= 3.75, considering κ_R _varying from 1 to10. We used the canonical genetic code for simulated sequences with 1.6 million codons and three sets of codon frequencies: equal (A to C), human (D to F) calculated from human protein-coding genes, and rice (G to I) calculated from rice protein-coding genes. ω = 0.3 (A, D, G), ω = 1 (B, E, H), and ω = 3 (C, F, I) were considered as representative values for purifying selection, neutral mutation, and positive selection, respectively.

In general, a better method should have relatively minimal deviations from real values with near infinite amount of data and within a reasonable range of all relevant parameters. In reality, we have to define both data in a limited way and parameter ranges within reasonable boundaries. In this exercise, we use ω = 0.3, 1, and 3 to represent negative, neutral, and positive mutations, respectively [3], and fix parameter *t *to 0.6 for initial assessment. Since genuine values for κ often range from 1.5 to 5, we always fix κ = 3.75 as typical. Considering that *γ*-MYN differentiates κ_Y _from κ_R_, we always fix one of them to 3.75 and allow the other varying from 1 to 10. We then analyze ω among data generated with YN, MYN, and *γ*-MYN against κ_R _(fixing κ_Y _= 3.75), using the three codon frequencies under different selective pressures (Figure 1A–I). We observed that *γ*-MYN produces less deviated ω from the standard data under negative selection as we perform analyses for different species. Although *γ*-MYN performs in a very similar way as MYN does, it is obviously better than YN under either positive or neutral selections. Since biased codon frequencies often have opposite effects as compared to the bias of transition/transversion rate ratio, ignoring codon frequency bias can lead to an overestimation of ω [13]. Using empirical data from human and rice, which represent distinct codon usages, we also did not detect any effect among different codon frequencies (Figure 1A–I). Since most of the evolutionary studies tend to calculate evolutionary rates between closely-related species, future research should focus more on the effect of different parameters and the improvement of calculations under negative selection.

### Effect of γ-distribution

MYN method assumes that different sites in a sequence evolve in the same way and at the same rate. It is obvious that such an assumption does not happen in the real world for most proteins and their genes. For instance, mutation rates are not the same in nuclear and organellar genomes among different species [39]. In addition, sequence variations among portions or domains of proteins mutate differently from a fixed mutation rate due to their specific structural and functional constraints for different genes under different selective pressures. Therefore, we introduced a parameter α in MYN method so that each substitution rate across sites is assumed to follow *γ*-distribution.

**Figure 2:**
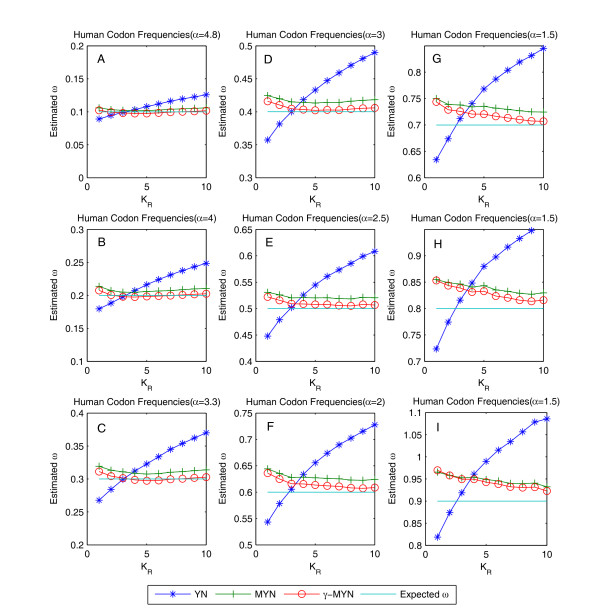
**Estimated ω when κ_Y _= 3.75 and κ_R _varies from 1 to 10 under negative selection**. We obtained better ω estimates by introducing parameter α when orthologous genes are under negative selection with ω varying from 0.1 to 0.9. The canonical genetic code was used for simulated sequences with 1.6 million human codons.

**Figure 3:**
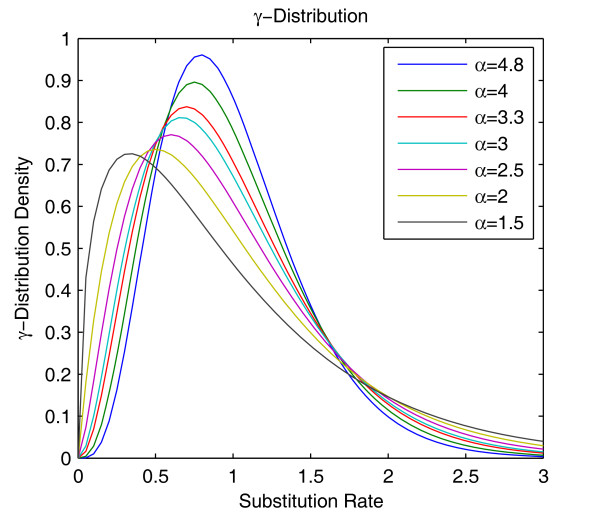
***γ *distribution density as a function of substitution rates at optimal α values**. We plotted different *γ *distribution densities as a function of substitution rates at optimal α values: (1) ω = 0.1, α = 4.8; (2) ω = 0.2, α = 4; (3) ω = 0.3, α = 3.3; (4) ω = 0.4, α = 3; (5) ω = 0.5, α = 2.5; (6) ω = 0.6, α = 2; (7) ω = 0.7, 0.8, and 0.9, α = 1.5. Note that each curve reaches its maximum and goes down with increasing substitution rates.

Since α is an unknown random variable and its variations may lead to changes of probability density of *γ*-distribution as well as deviations of *γ*-MYN method, we chose different parameters to force it to deviate from real values under different selective pressures (Figure 2). For a qualitative survey, the order of estimated values of ω, in the cases of κ_R _= 1, 2, and 3, is: YN <*γ*-MYN < MYN; the order of estimated values of ω for the rest cases, κ_R _= 4, 5, 6, 7, 8, 9, and 10, is: *γ*-MYN<MYN <YN. Furthermore, we observed that estimated ω do not change much as α varies when expected ω = 1 or 3, and *γ*-MYN again performs better when ω = 0.3 than it does when ω = 1 or 3. Because most calculated ω values indicate negative selection, and variation of α has stronger influence under negative selection, we analyzed the variation of ω in a range of 0.1 to 0.9 to evaluate the effect of α on ω. We obtained different optimal α values when ω varies from 0.1 to 0.9, and plotted different *γ *distribution densities (Figure 3). Each curve appears reaching its maximum and goes down with an increasing substitution rate. The peaks of the curves shift to the left and become lower in density when optimal α values decrease from 4.8 to 1.5; the decrease is attributable to the increase of ω (selective pressure) from 0.1 to 0.9. Furthermore, selective pressure shows significant effects on α, with an increase in the probability at the lowest and highest substitution rates across all sites. Because *γ*-MYN produces less biases than both YN and MYN do when ω varies from 0.1 to 0.9 under α = 4 (data not shown), we chose α = 4 as a typical value for our analyses.

### Effect of t

**Figure 4:**
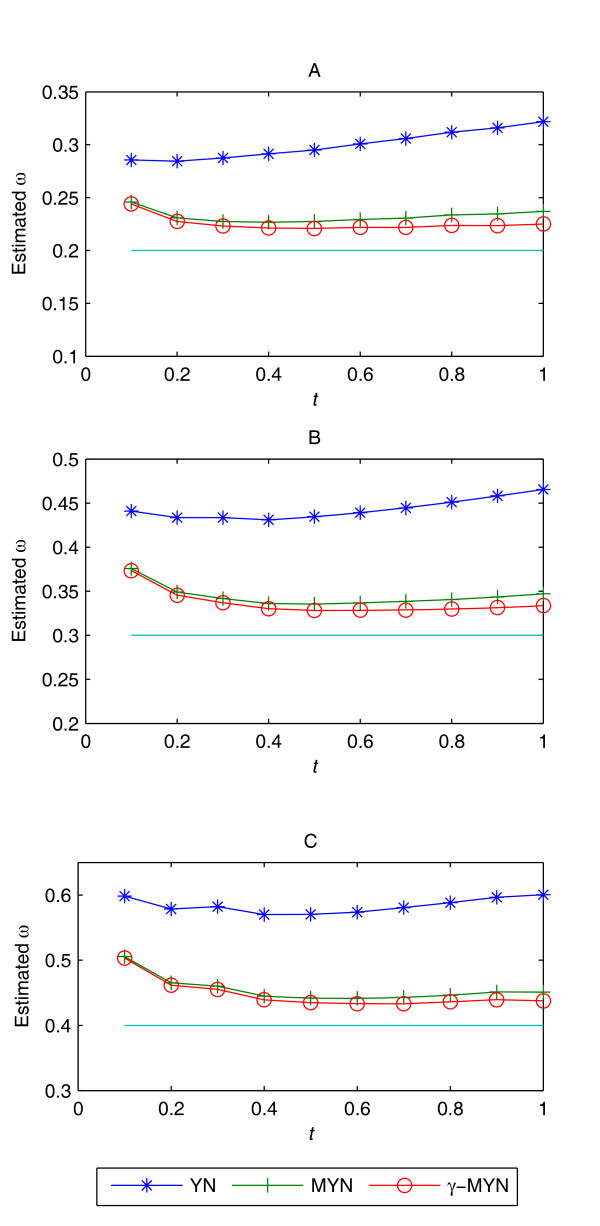
**The effect of *t *based on YN, MYN, and *γ*-MYN**. *γ*-MYN deviates less under the effect of *t *as compared to other methods. Both YN and MYN tend to overestimate ω. Since MYN and *γ*-MYN are two modified forms of YN, all datasets exhibit a similar trend. However, when *t *increases, *γ*-MYN performs better than the other two methods. Parameter values are (A) α = 4, κ_R _= 10, κ_Y _= 1, the expected value of ω = 0.2; (B) α = 4, κ_R _= 10, κ_Y _= 1, the expected value of ω = 0.3; and (C) α = 4, κ_R _= 10, κ_Y _= 1, the expected value of ω = 0.4.

The parameter *t *represents divergence time between two sequences. To test the effect of *t *on our method, we use human codon frequency (2,000 pairs of sequences with 400 codons for each case), and vary *t *from 0.1 to 1. Since *γ*-MYN does not change much in comparison with MYN under positive selection and neutral selection, we only consider the three obvious conditions of negative selection when κ_R _= 10 and κ_Y _= 1 are fixed: ω = 0.2, 0.3 and 0.4 (Figure 4). Although YN, MYN, and *γ*-MYN all have a nearly identical overall trend when *t *varies from 0.1 to 1, and they all tend to overestimate ω for negative selection, *γ*-MYN deviates less from the expected values. Despite the fact that *γ*-MYN also overestimates ω and the overestimation becomes less obvious as *t *increases, while the overestimation of both YN and MYN becomes severer.

### Effects of κ_R _and κ_Y_

**Figure 5:**
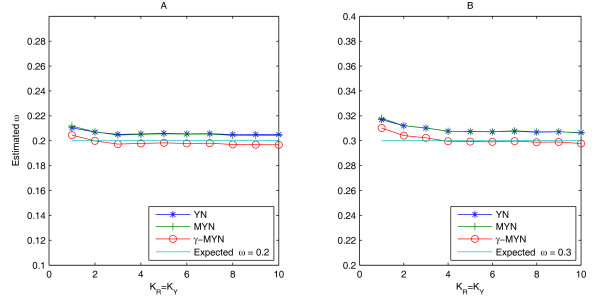
**The effects of κ_R _and κ_Y _based on YN, MYN, and *γ*-MYN**. We showed the effects of κ_R _and κ_Y _when ω = 0.2 (A) and 0.3 (B) were said to represent negative selection. The human codon frequency was used for the simulated sequences and α = 4 for both plots.

We used the same data (2,000 pairs of human codon sequences with 400 codons for each case) and methods to test the effects of κ_R _and κ_Y_. We plotted the average estimates of ω from YN, MYN, and *γ*-MYN methods against κ_Y _= κ_R _for the parameter combinations: the expected ω values vary as 0.2 and 0.3 when α = 4 (Figure 5). While the curves produce from YN and MYN methods superimpose each other when κ_R _(=κ_Y_) varies from 1 to 10, *γ*-MYN deviates clearly less from the expected ω. We found that *γ*-MYN still performs better than the other two methods, whereas MYN is degraded to YN when κ_Y _is equal to κ_R_. The result suggests that the assumption of variable substitution rates among different sites is necessary to Ka and Ks calculations.

### Effect of S%

Usually the effect of S% (the fraction of synonymous sites in a sequence) is considered as a factor of method evaluation. Changes in ω in relation to S% are often evaluated based on the effect of S% on the deviation of Ka and Ks. Therefore, an overestimated S% may give rise to underestimation of Ks and overestimation of Ka, resulting in overestimation of ω. Likewise, underestimation of S% may also lead to overestimation of Ks and underestimation of ω. It has been reported that S% has enormous influence on Ka and Ks under negative selection but has neglectable effect under positive selection [40]. We used human sequences to examine the effect of S% on *γ*-MYN (Table 2), fixing κ_Y _to 3.75. As κ_R _increases, the value of S% generated from our method exhibits minor fluctuations under different negative selections, when compared to that from YN. But the difference between *γ*-MYN and MYN is minute under this condition. In more details, the order of estimated values of S%, in the cases of κ_R _= 1, 2, and 3, is: YN < MYN <*γ*-MYN; in the rest cases, the order of estimated values of S%, when κ_R _= 5, 6, 7, 8, 9 and 10, is: MYN <*γ*-MYN < YN. We did not observe any obvious trend under the condition of κ_R _= 4. Therefore, *γ*-MYN is deemed insensitive to S% changes.

**Table 2:** S% Estimates under different negative selections based on YN, MYN, and *γ*-MYN

**Human Codon Frequencies**												
ω	α	Method	S (%)									
			
			κ_R _= 1	κ_R _= 2	κ_R _= 3	κ_R _= 4	κ_R _= 5	κ_R _= 6	κ_R _= 7	κ_R _= 8	κ_R _= 9	κ_R _= 10

0.1	4.8	YN	25.92	27.09	27.95	28.60	29.18	29.63	29.99	30.28	30.57	30.77
		MYN	29.03	28.88	28.78	28.66	28.56	28.50	28.42	28.34	28.29	28.16
		*γ*-MYN	29.12	28.94	28.83	28.70	28.59	28.53	28.45	28.36	28.31	28.18

0.2	4	YN	25.91	27.07	27.92	28.62	29.22	29.66	30.04	30.36	30.65	30.87
		MYN	29.00	28.87	28.69	28.57	28.53	28.43	28.30	28.26	28.21	28.15
		*γ*-MYN	29.10	28.95	28.75	28.62	28.57	28.47	28.33	28.28	28.23	28.16

0.3	4	YN	25.90	27.06	27.94	28.65	29.19	29.63	30.04	30.38	30.65	30.90
		MYN	29.05	28.87	28.70	28.57	28.43	28.33	28.26	28.19	28.14	28.06
		*γ*-MYN	29.14	28.95	28.76	28.61	28.47	28.36	28.28	28.21	28.15	28.08

0.4	3	YN	25.85	27.06	27.96	28.65	29.20	29.66	30.05	30.37	30.64	30.90
		MYN	28.96	28.84	28.70	28.55	28.44	28.35	28.27	28.22	28.16	28.10
		*γ*-MYN	29.08	28.94	28.78	28.62	28.50	28.40	28.31	28.25	28.18	28.11

0.5	2.5	YN	25.88	27.05	27.95	28.66	29.23	29.66	30.04	30.35	30.64	30.86
		MYN	28.97	28.81	28.68	28.56	28.47	28.36	28.29	28.18	28.15	28.05
		*γ*-MYN	29.12	28.93	28.77	28.63	28.53	28.41	28.33	28.21	28.17	28.07

0.6	2	YN	25.89	27.05	27.92	28.66	29.20	29.66	30.02	30.34	30.61	30.88
		MYN	28.99	28.81	28.64	28.58	28.45	28.37	28.27	28.18	28.10	28.09
		*γ*-MYN	29.19	28.96	28.77	28.67	28.52	28.43	28.31	28.22	28.13	28.12

0.7	1.5	YN	25.87	27.05	27.93	28.63	29.21	29.65	30.02	30.33	30.63	30.86
		MYN	28.94	28.79	28.65	28.54	28.47	28.36	28.27	28.16	28.12	28.05
		*γ*-MYN	29.21	28.99	28.82	28.67	28.57	28.44	28.33	28.21	28.16	28.08

0.8	1.5	YN	25.86	27.05	27.96	28.62	29.20	29.65	30.02	30.33	30.61	30.86
		MYN	28.93	28.79	28.69	28.52	28.48	28.37	28.27	28.17	28.11	28.05
		*γ*-MYN	29.19	29.00	28.86	28.65	28.58	28.45	28.34	28.22	28.14	28.08

0.9	1.5	YN	25.87	27.06	27.96	28.65	29.20	29.66	30.01	30.33	30.61	30.85
		MYN	28.91	28.80	28.67	28.57	28.47	28.38	28.27	28.17	28.08	28.04
		*γ*-MYN	29.18	29.01	28.84	28.69	28.58	28.46	28.34	28.22	28.12	28.07

### Effect of sequence lengths

The length of homologous genes subjected to an analysis usually varies in actual calculation. In order to evaluate the effect of variable sequence lengths, we use two groups of simulated rice sequences under the conditions of (1) ω = 0.2, κ_R _= 10, κ_Y _= 1, *t *= 0.6, and α = 4; and (2) ω = 0.3, κ_R _= 10, κ_Y _= 1, *t *= 0.6, and α = 4. We then calculate the average estimated ω when the number of codons varied from 100 to 1,000 (Table 3). It appears that all three methods overestimate ω regardless the number of codons in the datasets. In particular, despite the fact that all three methods give rise greater biases for shorter sequences (<300 codons), *γ*-MYN performs better than the other two methods. We also found that the performance of *γ*-MYN is getting better faster than the other two methods as the number of codon increases.

**Table 3:** Average ω estimates calculated based on YN, MYN and *γ*-MYN.

**Rice Codon Frequencies (α = 4)**						
Number of codons	ω = 0.2			ω = 0.3		
	
	YN	MYN	*γ*-MYN	YN	MYN	*γ*-MYN

100	0.308	0.245	0.235	0.458	0.364	0.352
200	0.305	0.230	0.222	0.450	0.341	0.332
300	0.294	0.219	0.210	0.435	0.325	0.316
400	0.290	0.215	0.207	0.426	0.317	0.308
500	0.294	0.216	0.208	0.430	0.317	0.308
600	0.291	0.214	0.206	0.427	0.316	0.307
700	0.290	0.213	0.205	0.424	0.313	0.305
800	0.290	0.212	0.205	0.424	0.313	0.305
900	0.288	0.212	0.204	0.422	0.312	0.303
1000	0.287	0.210	0.203	0.421	0.310	0.302

Note: The parameters used are κ_R _= 10, κ_Y _= 1, *t *= 0.6, and α = 4. ω = 0.2 and ω = 0.3 are used separately to represent purifying selection. ω values are averaged over 2,000 pairs of simulated sequences.

### Testing real data

**Figure 6:**
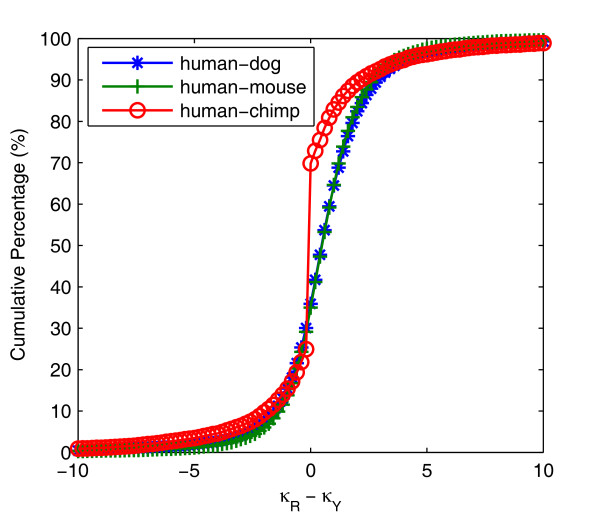
**Cumulative percentage of κ_R _- κ_Y_ for human-dog, human-mouse and human-chimp orthologs at a bin size of 0.2**. We divided the x-axis into 100 bins and plotted the cumulative percentage of κ_R _- κ_Y _from the orthologous genes of human-dog, human-mouse and human-chimp.

We used three ortholog datasets for the test, 14,323 from human-dog, 16,066 from human-mouse, and 12,351 from human-chimp. For a more comprehensive display, we examined the cumulative percentage of κ_R_-κ_Y _(Figure 6), showing different transitional substitutions with unequal frequencies. For example, the cumulative percentages for κ_R _- κ_Y _> 0.4 for human-dog, human-mouse and human-chimp orthologs are 52.27%, 52.66%, and 24.47% and those for κ_R _- κ_Y _< -0.4 are 25.36%, 24.31%, and 21.87%, respectively. In the rest cases, for |κ_R _- κ_Y_| ≤ 0.4, they are 22.37%, 23.02%, and 53.66% for the three ortholog groups. We found that the value for human-chimp is more than twice as much as that of human-dog (or human-mouse), and the reasons are attributable to a close evolutionary relationship between human and chimpanzee [41].

To evaluate the performance of *γ*-MYN, we compared a set of values for several key parameters (S%, Ka, Ks, and ω) generated with *γ*-MYN and three other selected methods in a straightforward way, considering three cases of κ_R _- κ_Y _> 0.4, κ_R _- κ_Y _< -0.4, and |κ_R _- κ_Y_| ≤ 0.4 (Table 4). We chose the value of 0.4 as a threshold so that the three cases can stand for three groups of κ_R _under the condition of κ_Y _= 3.75: (1) κ_R _= 5, 6, 7, 8, 9 and 10; (2) κ_R _= 1, 2, and 3; (3) κ_R _= 4. Other than YN and MYN, we also used a maximum likelihood method proposed by Goldman and Yang (denoted as GY) [33].

**Table 4:** Proportions of synonymous sites (S%) and estimates of Ka, Ks and ω

Method	κ_R _- κ_Y _> 0.4				κ_R _- κ_Y _< -0.4				|κ_R _- κ_Y_| ≤ 0.4			
	
	S%	Ka	Ks	ω	S%	Ka	Ks	ω	S%	Ka	Ks	ω
human-dog orthologs												
GY	24.80%	0.0691	0.4936	0.1483	24.72%	0.0723	0.4676	0.1639	24.34%	0.0874	0.5347	0.1704
YN	25.09%	0.0674	0.4900	0.1531	24.96%	0.0700	0.4750	0.1695	24.27%	0.0928	0.5412	0.1746
MYN	23.88%	0.0664	0.5748	0.1361	26.16%	0.0711	0.4495	0.1800	24.22%	0.0939	0.5596	0.1727
γ-MYN	23.93%	0.0681	0.6462	0.1266	26.28%	0.0733	0.4962	0.1713	24.29%	0.0962	0.6227	0.1620
												
human-mouse orthologs												
GY	25.86%	0.0901	0.7163	0.1291	25.65%	0.0961	0.7118	0.1390	25.52%	0.1128	0.7422	0.1543
YN	26.07%	0.0877	0.7002	0.1344	25.69%	0.0923	0.6904	0.1465	25.31%	0.1091	0.7439	0.1564
MYN	24.83%	0.0863	0.8321	0.1157	26.93%	0.0940	0.6527	0.1566	25.24%	0.1090	0.7734	0.1526
γ-MYN	24.91%	0.0894	0.9501	0.1058	27.10%	0.0980	0.7390	0.1468	25.35%	0.1140	0.8934	0.1398
												
human-chimp orthologs												
GY	25.47%	0.0273	0.0663	0.5118	25.18%	0.0262	0.0579	0.5685	25.98%	0.0228	0.0420	0.4364
YN	25.79%	0.0302	0.0646	0.5237	25.33%	0.0297	0.0564	0.5790	23.81%	0.0506	0.0595	0.3893
MYN	24.34%	0.0299	0.0719	0.4792	26.87%	0.0307	0.0516	0.6312	23.72%	0.0493	0.0601	0.3863
γ-MYN	24.34%	0.0305	0.0741	0.4768	26.89%	0.0324	0.0530	0.6309	23.72%	0.0515	0.0626	0.3847

The results showed a few interesting trends. First, GY performs in a similar way as YN does as compared to MYN and *γ*-MYN; it is consistent with our previous simulation results, as they share a common consideration of transition/transversion rate bias and nucleotide frequencies bias [12,42]. Second, the trends of ω estimates with the three methods, YN, MYN and *γ*-MYN, are consistent with our simulation results. In the cases of |κ_R _- κ_Y_| ≤ 0.4 and κ_R _- κ_Y _> 0.4, when κ_R _= 4, 5, 6, 7, 8, 9, and 10, the order of estimated values of ω is: *γ*-MYN < MYN < YN. When confined to κ_R _- κ_Y _< -0.4, when κ_R _= 1, 2, and 3, YN underestimates ω and MYN overestimates ω as compared to *γ*-MYN. Taking ω estimates as an example, they are 0.1695, 0.1800, and 0.1713 for human-dog orthologs, 0.1465, 0.1566, and 0.1468 for human-mouse orthologs, and 0.5790, 0.6312 and 0.6309 for human-chimp orthologs, calculated with YN, MYN, and *γ*-MYN, respectively. These findings are in agreement with our simulation studies. Third, the orders of S% estimates with the three methods (YN, MYN and *γ*-MYN) are also consistent with our simulation results. For example, when κ_R _- κ_Y _> 0.4, κ_R _= 5, 6, 7, 8, 9 and 10, YN overestimates S% and MYN underestimates S% as compared to *γ*-MYN. In the case of κ_R _- κ_Y _< -0.4, when κ_R _= 1, 2, and 3, the order of estimated values of S% is: YN < MYN <*γ*-MYN. Fourth, we took one gene as an example to show the outperformance of our new method over others. Among the human-chimp orthologs, ω values of an immunoglobulin interleukin-1-related receptor (NP_068577) are listed as 1.02406, 0.622611, 0.59999, and 0.843755, when *γ*-MYN, MYN, YN, and GY are used for the calculation, respectively. Obviously, only *γ*-MYN is able to detect positive selection for this gene, and others failed. This gene has been studied previously based on a population genetics analysis of extended-haplotype-homozygosity in Northeast Asians, and a possible positive selection scheme was proposed for it [43]. This result is in accordance with the result of large-scale scanning on positively selected genes between human and chimpanzee genomes [44,45].

### Program availability and performance

A C++ program implementing *γ*-MYN method is included in the updated KaKs_Calculator [42], available upon request. And we tested the running time with YN, MYN, *γ*-MYN, and GY, using the three testing datasets (14,725 human-dog, 16,368 human-mouse, and 15,646 human-chimp gene pairs). Table 5 shows the time consumption for each method to compute Ka/Ks ratios from the three datasets and their average running time. On average, *γ*-MYN takes 600 folds less time than GY does, and YN, MYN, and *γ*-MYN perform similarly in time consumption. We believe that *γ*-MYN may become a useful tool for large-scale studies, when ML-based methods (such as GY) are deemed time-consuming.

**Table 5:** Timing comparisons on YN, MYN, γ-MYN and GY methods

Method	Time Required, seconds (hr:min:sec)			Average
		
	human-dog	human-mouse	human-chimp	
YN	332(0:5:32)	389(0:6:29)	280(0:4:40)	334(0:5:34)
MYN	529(0:8:49)	641(0:10:41)	396(0:6:36)	522(0:8:42)
γ-MYN	533(0:8:53)	639(0:10:39)	395(0:6:35)	522(0:8:42)
GY	154309(42:51:49)	233899(64:58:19)	602381(167:19:41)	330196(91:43:16)

The analyses were performed on IBM HS21, INTEL 5335 2.0GHz, memory of 16GB, ROCKS LINUX 4.3 X86-64 platform.

## Discussion

### Why should we continue developing Ka/Ks methods?

A major limitation of Ka/Ks methods, mentioned in literatures, is their poor ability for detecting positive selection (adaptive selection) [6,46-52]. To detect positive selection at sites requires that ω value averaged over all branches is >1 and to detect positive selection along lineages requires ω value averaged over all sites is >1 [6]. Therefore, Ka/Ks methods are only useful to weight average selection pressure over sites and branches. They may not be able to detect positive selection for some highly conserved proteins that are mostly invariable but become fragile when a single site alters. Other detrimental cases include transmembrane domains where high variability may not change its physiochemical property. To overcome the weakness, there have been methods developed, such as Likelihood Ratio Test (LRT) [53-56] implemented in PAML [57] and Hyphy software [58], to identify positive selection [45,59-61], which tend to be qualitative. An obvious pitfall of these methods is that they do not weigh the relative degree of two genes under negative selection. Ka/Ks methods can perform better in this regard [38,62-64] as they tend to be more quantative. In addition, Ka/Ks methods can be readily extended from our current work to detect the sequence alternations that lead to protein structure changes and positive selection, in combination with other techniques, such as ancestral sequence reconstruction [65-67] and primary [68,69] or tertiary windowing [70,71]. Therefore, we believe that the two different lines of methods (LRT-like methods and Ka/Ks methods) should also be useful under appropriate conditions.

### Why should we introduce new parameters?

With the introduction of the parameter *α*, our method *γ*-MYN shows significant improvements when compared with the other two related methods in both simulation and tests on real data. As *γ*-MYN assumes that evolutionary rate at each site follows *γ*-distribution, we found that the parameter α has observable effects under different evolution rates. For instance, when ω >= 1, *γ*-MYN remains stable. We also observed that selective pressure can overwhelm the variable substitution rates across sites and it becomes the most influential factor when increasing dramatically. Therefore, when we consider strong positive selection and neutral evolution, the effect of variable substitution rates across sites can be somewhat neglected. In addition, more parameters often lead to increase of complexity of an algorithm, resulting in the decrease of efficiency. However, we hold the view that proper introduction of parameters is worthwhile.

It has been noticed that the majority of the evolutionary selections are actually negative in nature, and the statement is confirmed by our analyses on real data. When ω varies from 0.1 to 1, we selected optimal *α *to minimize biases and found that *γ*-MYN is very sensitive when *α *changes under negative selection. Furthermore, the optimal α becomes smaller when ω becomes larger under negative selection, and the effects of various substitution rates across sites become evident under negative selection, emphasizing the importance of our method in the calculation under negative selection.

As to γ-Tamura-Nei model, it usually leads to higher variations, especially in phylogeny analyses. However, we found some multi-level observations in both simulation and real data testing (see in additional file 1). The difference of variations of ω between YN and γ-MYN (or MYN) seems to be correlated to the value of κ_R _- κ_Y_, when results in Tables S2-S6 were examined together. For instance, when κ_R_-κ_Y _< 0, ω values vary more when γ-MYN is used than YN is. As κ_R_-κ_Y _increases, the ω variation values from γ-MYN decrease, leading to lower numbers than those of YN. The variations of ω calculated with *γ*-MYN are slight higher than those yielded from MYN in most cases but not all. These results reflect the distinction between the usage of γ-Tamura-Nei model (or Tamura-Nei model) in KaKs computation and that of phylogeny reconstruction.

### How the variable substitution rates influence the Ka/Ks calculations?

We begin our discussion with how *α *parameter in *γ*-MYN improves MYN method. It is known that ignoring rate variation among sites leads to underestimation of both the sequence distance and the transition/transversion rate ratio κ (both κ_R _and κ_Y_) [4]. κ is used not only in estimating S and N but also in generating a transition probability matrix for estimating S_d _and N_d_. If we derive an approximate formula for ω = Ka/Ks ≈ (N_d_/N)/(S_d_/S) (the symbol of "≈" is used to emphasize the absence of correction for multiple substitutions), ω is composed of two parts: N_d_/N and S_d_/S. For purifying selection, synonymous substitutions occur more frequently than nonsynonymous ones so we should only focus on Ks (S_d_/S). Since κ decrease is related to the reduction of substitution rate between two codons, underestimation of κ leads to underestimation of S_d_. In addition, nucleotide transitions between two codons are more likely to be synonymous especially at the third codon positions, underestimation of κ leads to underestimation of S. However, the influence of κ on S_d _is significantly stronger than that on S. As a consequence, an underestimated κ, when used in MYN, may give rise to underestimation of S_d_/S, resulting in overestimation of ω as compared with *γ*-MYN. Our theoretical deductions are consistent with both simulation (Figure 2) and real data (Table 4).

In addition, we found the optimal *α *values fall between 1 and 5 (Figure 3). In these cases, the distribution of gamma values is bell-shaped, meaning that most sites have intermediate rates around 1 whereas a few sites have either very low or very high rates [4]. When selection pressure increases, the number of sites of intermediate rates decreases (Figure 3). In particular, when α approaches infinity, the distribution diminishes into the model of a single rate for all sites, which is used in MYN method. If *α *≤ 1, the distribution has a highly skewed L-shape, suggesting that most sites have either very low rates of substitution or are nearly "invariable" with possible substitution hotspots. Furthermore, estimates of *α *from real data in many species over multiple sequences show increases from 0.26 to 3.0, and this relatively wide window allows us to explore the spectrum of different substitution rates over different sites [4]

### Applications of our new method

Divergence time (*t*) is another parameter important for the estimation of Ka and Ks. When divergence time reaches the extremes, the compared sequences among genes often vary considerably and their corresponding protein structures may changed over greater evolutionary time scale. Therefore, under such conditions it may become meaningless to calculate the substitution rates of such genes. However, most methods for calculating Ka and Ks use homologous genes for estimating substitution rates among closely-related species or within close lineages, and observable selections are mostly negative. Since our method *γ*-MYN has better performance than other methods when ω < 1, it provides a useful alternative for more comprehensive Ka and Ks calculations.

Our past work has testified that methods for estimating Ka and Ks should be used cautiously and one should not draw simple conclusions on gene evolution from Ka and Ks analyses based on a single method. Therefore, we recommend a method based on model selection and model averaging [12,42], and *γ*-MYN has just brought a new choice into such endeavours. Our method does not challenge other methods such as GY (Goldman-Yang) method, a typical maximum likelihood (ML) that has been always considered to be the method of choice [13,33]. It has been suggested in the literature that GY and YN both give rise to similar estimates on Ka and Ks primarily due to the fact that they both take account of major dynamic features of DNA sequence evolution, including transition/transversion rate and nucleotide/codon frequency biases [12,42]. As *γ*-MYN performs better than other methods under certain conditions, despite the fact that its advantages seemed less obvious under other conditions, we believe that *γ*-MYN may become a useful tool for large-scale sequence analysis when ML-based methods are deemed time-consuming.

## Conclusion

We compared *γ*-MYN with two other methods, YN and MYN, by examining long sequences, performing computer simulations, and analyzing real datasets. Since neglecting the variation of substitution rates among different sites may lead to biased estimates, our new method has minimal deviations when parameters vary within normal ranges defined by empirical data. *γ*-MYN performs better when genes are under strong purifying selection and comparable to the other two methods when genes are under positive selection or remain neutral. In addition, we showed that biased estimates of Ka and Ks primarily originate not only from biased estimates of κ–or both κ_R _and κ_Y_–but also from the neglect of variable substitution rates.

## Methods

### Mutation model

In Markov-chain models of codon substitution, the codon triplet is considered as the unit of evolution, and a Markov chain is used to describe substitutions from one codon to another codon [4]. In detail, the state space of the chain is the sense codons with regard to the canonical genetic code. Stop codons are not allowed inside a functional protein and are not considered. Although there are several mutation (substitution) models that take different sequence variation features into account, in this report we limit our discussions to the Tamura-Nei Models [24] (see Table S1 in additional file 2 for details).

*γ*-MYN also needs a transition probability matrix similar to YN and MYN. We assigned the substitution rate q_ij _from any codon i to j (i ≠ j) to generate a transition probability matrix as follows:

(1)

The diagonal elements of the transition probability matrix, *Q *= {*q*_*ij*_}, are determined based on the mathematical requirement that the row sums equal to zero. The matrix is normalized with the result that the sum over non-diagonal terms is 1.

### Estimating κ_R _and κ_Y_

To generate the transition probability matrix, we need to estimate κ_R _and κ_Y_. Similar to YN and MYN, we calculated four nucleotide frequencies (g_T_, g_C_, g_A_, g_G_), proportions of transitional differences between purines (T_R_), and between pyrimidines (T_Y_), and the proportion of transversional differences (V) from compared sequences:

(2)

where g_R _= g_A _+ g_G _and g_Y _= g_T _+ g_C_. Note that α is the square of the inverse of the variation coefficient in the gamma function.

We then used equation 3 to estimate κ_R _and κ_Y_.

(3)

(4)

The detailed procedures for deducing κ_R _and κ_Y _were summarized in additional file 2. We also made other modifications accordingly, such as using κ_R _and κ_Y _to estimate S and N, generating relevant transition probability matrix (Equation 1), considering different transitional evolution pathways to count S_d _and N_d_, and correcting for multiple substitutions when estimating Ka and Ks (Equation 4; [24]).

### The algorithm

When compared to MYN and YN, our *γ*-MYN method considers that the rate of nucleotide substitution *λ *approximately follows *γ*-distribution. In fact, if the rate of nucleotide substitution *λ *is the same for all sites considered, the model becomes the model that is used in MYN.

*γ*-MYN uses an iterative approach to estimate Ka and Ks. Before iteration, *γ*-MYN computes nucleotide frequencies (regarding to the three codon positions), κ_R_, κ_Y_, S, and N from the sequences to be analyzed. Based on the F3 × 4 model [36], codon frequencies are calculated by multiplying each nucleotide frequencies. κ_R _and κ_Y _are estimated from four-fold degenerate sites at the third codon position and non-degenerate sites. S and N are calculated by using κ_R_, κ_Y_, and codon frequencies. *γ*-MYN chooses initial values for *t *and ω as starting point for iteration. It generates a transition probability matrix that represents substitution probabilities from one codon to another by using ω, *t*, κ, and codon frequencies. This transition probability matrix is subsequently used to deduce S_d _and N_d _and for new estimates of ω and *t*. *γ*-MYN repeats the calculation for another transition probability matrix, until the algorithm converges.

### Comparative analysis on Ka and Ks estimations

We used simulated sequences generated from hypothetical common ancestral sequences for our comparative analysis by randomly choosing codons (61 excluding stop codons) from the ancestral sequences according to codon frequencies that were derived from three empirical datasets: (1) equal codon frequencies [13], (2) human codon frequencies deduced from 39,420 human protein-coding genes from ENSEMBL database (Release 35; [37]) and (3) rice codon frequencies deduced from 19,079 rice protein-coding genes [38] (see in additional file 1).

In addition to codon frequencies, we also have to fix or choose ranges of other parameters for the simulation, including sequence length, divergence time (*t*), two ratios of transitional rate between purines (κ_R_) and between pyrimidines (κ_Y_) to transversional rate, and selective pressure ω. Although ω varies from gene to gene, ω = 1 and 3 can be regarded as "typical values" for neutral mutation and positive selection, respectively [3,13,67], which are observable from real datasets. Since most calculated ω values indicate negative selection and variation of parameter α has stronger influence under negative selection, we analyzed the variation of ω in a range of 0.1 to 0.9 for the evaluation of effects of α on ω. To accurately examine the effect of one parameter and to avoid stochastic errors arising from other factors, we generated 2,000 pairs of sequences. Three orthologous gene sets were downloaded from NCBI's HomoloGene database (Build 61), which contained 14,725 human-dog, 16,368 human-mouse, and 15,646 human-chimp gene pairs [72]. We considered "NA" occurrence (in any of Ka, Ks or ω) as unreliable data and filtered the orthologous pairs (extremes in sequence homology) that have such labels, and 14,323 human-dog, 16,066 human-mouse, and 12,351 human-chimp gene pairs were remained. The datasets were used for comparing *γ*-MYN with other methods.

## Competing interests

The authors declare that they have no competing interests.

## Authors' contributions

DPW deduced formulas and analyzed the data. DPW, HLW and SZ drafted the manuscript. SZ programmed this new method. DPW, HLW and SZ carried out computer simulations. JY supervised the research and revised the manuscript. All authors read and approved the final manuscript.

## Reviewers' comments

### Reviewer 1

Kateryna Makova (assisted by Mr. Chungoo Park), Center for Comparative Genomics and Bioinformatics, Department of Biology, 305 Wartik Lab, Penn State University, University Park, PA, 16802

In this paper, the authors suggest a new algorithm, called γ-MYN, to accurately estimate nonsynonymous and synonymous substitution rates (Ka and Ks) of protein-coding DNA.

The γ-MYN considers the variation of substitution rates among different sites in a sequence, which is overlooked by existing methods, and shows that their unequal substitution rates affect Ka and Ks.

#### Specific comments

1. Authors should highlight standard deviations of ω (as well as Ka and Ks) in all tests and show significance of difference in all comparisons.

#### Authors' response

*Agreed. We added the standard deviations for the calculations of ω, Ka, Ks and S% in key analyses in the supplementary materials*.

2. To represent distinct codon usages by two genomes (human and rice), authors should reveal their codon usage difference in the paper.

#### Authors' response

*Agreed. We added the tables for human codon frequencies and rice codon frequencies to the supplementary materials*.

3. Why were some tests carried out for rice genome, while the other tests for human genome? For example, rice sequences (and not human sequences) were used to study the effect of sequence lengths.

#### Authors' response

*Human and rice were selected to represent the genomes of animals and plants in this research. In the part of "testing the effect of codon frequencies", we did not observe any significant differences when use different codon frequencies. Under most conditions, we chose to use human codon frequencies*.

4. How are optimal α values statistically determined in each test?

#### Authors' response

*We added the details to the supplementary materials*.

5. The authors find that human-chimp orthologs have higher ω values than human-dog (or human-mouse) orthologs and thus claim that many genes (i.e., 1075 from human-chimp orthologs; 25 and 14 for human-dog and human-mouse, respectively) evolve under strong positive selection. However, this is incongruent with Bakewell et al. (2007; PMID: 17449636) in terms of the number of genes under positive selection and ω values. Authors should discuss the difference in detail.

#### Authors' response

*Bakewell et al*[44]*indeed identified two gene sets, 154 and 233 positive selected genes or PSG for human and chimpanzee lineages, respectively, the authors also claimed that the branch-site likelihood method was not able to detect all PSGs according to their results from computer simulation. Therefore, we speculate that both their and our estimates about the numbers of PSG are not thorough enough, limited by our methodology. In our calculation, we did not distinguish the two lineages, only computed the average values across the lineages, and did not consider the common ancestor of human and chimpanzee. We believe that only an in-depth population genetic analysis may resolve such issues. As far as ω is concerned, our methods are based on the raw definition of Ka/Ks and their methods are based on branch-site test. Interestingly, most of the functional categories of PSG genes in both studies overlap significantly, especially in "protein metabolism & modification" and "stress response and immunity"*.

6. Regardless of the number of codons and divergence time (t), why do all three methods (γ-MYN, MYN, and YN) overestimate ω values?

#### Authors' response

*We believe that this is perhaps due to similar assumptions with the same parameter settings (i.e. κ_R _= 10, κ_Y _= 1) in the two calculations. However, the differences are also obvious as we tried to demonstrate throughout our manuscript*.

7. It is not clear how the "unreliable data" from three orthologous gene sets were excluded.

#### Authors' response

*We addressed this in our revised manuscript*.

8. It is not clear how multiple splicing variants for each gene were handled to obtain codon frequencies.

#### Authors' response

*We did not distinguish splicing variants in our study. In other words, we assumed that codon frequencies are the same for all possible alternatively spliced forms*.

9. It is surprising that a set of human-dog gene pairs took longer to compute Ka/Ks ratios than that of human-chimp gene pairs(533 sec versus 395 sec for γ-MYN), even though the number of human-dog gene pairs was smaller than that of human-chimp gene pairs (14,725 versus 15,646).

#### Authors' response

*As the sequence variation of individual gene pairs governs the time for the calculation, the required time is not proportional to the number of gene pairs but the number of effective sites*.

### Reviewer 2

David A. Liberles, Department of Molecular Biology, University of Wyoming, Laramie WY 82071, USA

"γ-MYN: A new algorithm for estimating Ka and Ks with consideration of variable substitution rates" by Wang, Wan, Zhang, and Yu describes a more model rich version of the original Yang and Nielsen 2000 model [13]. Previous work added parameters to differentiate between purine transitions and pyrimidine transitions [14]. The current work adds a gamma distribution on top of the previously described work.

The stepwise addition of parameters to the Yang and Nielsen approach reflects an attempt to add increasing layers of biological realism. Differentiating between purine and pyrimidine transitions is driven by potential underlying forces like codon bias to the extent that it is correlated across codons in a gene and the chemistry (specificity) of DNA damaging agents, DNA polymerase, and DNA repair enzymes (see [73] for example). The biological link to the gamma distribution is somewhat less clear in the way that it has been applied. Nucleotide sequences as well as amino acid sequences typically show support for a gamma distribution characterizing rates across sites. At the nucleotide level, this is typically related to two components: differences in substitution rate in the different codon positions due to the nature of the genetic code as well as amino acid level constraint on the protein. The former category is already modeled with ω, potentially creating some degree of redundancy between the ω and α parameters. Modeling α at the amino acid level (translated codons) would not suffer from the redundancy and likely accounts for the improvement in performance by γ-MYN.

#### Authors' response

*We agree with reviewer's excellent explanation why γ-MYN is capable of improving the performance of omega calculation. Modeling α at the amino acid level avoids suffering from the parameter redundancy especially when genes are subjected to negative selection as in one of our unpublished results, we found that the interplay of α parameter and other evolutionary features may show some degrees of redundancy*.

The authors show the improved performance of γ-MYN on simulated data, where the correct answer is known. This is necessary but not sufficient to support the use of additional parameters on real data, as the model is effectively recovering itself on simulated data. The authors do apply γ-MYN to mammalian comparative genomic data. However, it should be possible to evaluate the likelihood of the sequence data given the model and its parameterization for γ-MYN compared to simpler models and to evaluate the performance of the models with AIC, even if the methods are approximate rather than proper likelihood estimates.

#### Authors' response

*In our previous work, we did incorporate AIC to KaKs calculations *[42]*and found that the selected models in the calculation did not depend on combinations of various parameters. We speculate that γ-MYN perhaps may not be the best choice under certain conditions, when the smallest AIC is considered as the criteria. We will incorporate AIC into our new model and the updated KaKs Calculator (the software through model selection and model averaging)*.

A more minor point is that the authors suggest that approximate methods need to average over all sites or all branches. Based upon earlier work using ancestral sequence reconstruction coupled with counting methods [65-67], approximate methods have been developed or can easily be extended from current work based upon primary windowing to detect selective sweeps [68,69] and tertiary windowing to detect structural covariation leading to positive selection [70,71]. This should probably be discussed when discussing the power of these methods.

#### Authors' response

*We expanded our discussion into this issue. The power of detecting positive selection in KaKs methods certainly can be enhanced by introductions of ancestral sequence reconstruction and sliding windows. However, it is still an interesting question as which one is better when compared to the LRT methods*.

Further development of models based upon mechanistic molecular and biological underpinnings is always a welcome addition to the literature. A number of problems from multiple sequence alignment to amino acid-based phylogeny to problems in detecting positive selection suffer from the divorce of common models from underlying processes. Well-performing mechanistic models will be broadly applicable across bioinformatics.

#### Authors' response

*We fully agree with this comment. In the real world, sequence analysis can be complex and difficult. Therefore, models considering more biological parameters lay foundations for broader applications, especially in the field of molecular evolution (phylogeny tree reconstruction and mechanics of evolution dynamics)*.

### Reviewer 3

Zhaolei Zhang, Banting & Best Dept. of Medical Research (BBDMR), Department of Molecular Genetics, University of Toronto, 160 College St., Room 608, Donnelly CCBR Building, Toronto, ON M5S 3E1, Canada

"**γ (gamma)-MYN: A new algorithm for estimating Ka and Ks with consideration of variable substitution rates**"

Authors: Da-Peng Wang, Hao-Lei Wan, Song Zhang, and Jun Yu.

#### General comments

This manuscript describes a new method to estimate the ratio of Ka/Ks taking into account the evolutionary rate variation. Ka/Ks ratio is commonly used as an indicator of selective pressure acting on protein-coding genes. Current methods mostly use simplified substitution models, which may have effect on the estimation of Ka/Ks. Here, based on their previous work, the authors present a new method that the evolutionary rates across sites are modeled by a gamma distribution. Using both simulated and real data, the authors show that the new method performs better than current methods under some conditions.

The novelty of this manuscript is that this is the first Ka/Ks estimation method that considers the rate variation among sites. It is an important contribution to the scientific communities that use Ka/Ks in their research, and likely will open avenues for new researches in this area.

#### Specific comments

I found the overall writing is clear, albeit a little verbose at some places.

#### Authors' response

*We revised the manuscript again for clarity*.

#### Concern of overfitting

Can the authors address the concern of over-fitting by introducing additional parameters?

#### Authors' response

*We would like to address this issue as it has been a major concern all along. First, more complex models (e.g. allowing for the correlation of substitution rates at adjacent sites and thus parameter-rich) used in phylogenetic analyses usually produce similar results to simple gamma models *[31]. *Second, the opposite is usually true also as some methods with simple assumptions often lead to similar results over complex ones. For instance, Nei *[7]*developed a simple method (giving no weights to different types of codon substitutions) that gives essentially the same results as those more complicated methods (such as giving different evolutionary pathways different weights). Third, more parameters usually lead to higher sensitivities to sequence variations albeit untenable in certain cases, especially when testing some real data. However, setting more parameters, especially by estimating their optimal ranges, we should be able to assess the relationships between parameters and the characteristics of the real data as well as tradeoffs between parameters and models*.

#### Specific examples

Is it possible for the authors to show one specific example (a gene) that the new method out-performs other methods, i.e. the conclusion is more biologically relevant?

#### Authors' response

*Limited by the manuscript length, we decided to show our analysis on one real gene in the "testing real data" section*.

### Reviewer 4

Shamil Sunyaev, Harvard Medical School, Boston, MA, United States

This manuscript presents a new method to compute Ka and Ks. The authors incorporated Tamura-Nei model into the Yang-Nielsen approach. This extension of the method would be of interest to experts in molecular evolution. I have a few comments and suggestions.

1) The manuscript would benefit from a much clearer justification of the method and discussion of its applicability. Tamura-Nei model was developed for the control region of the mitochondrial genome. Is the model with the uniform selective constraint (reflected by parameter omega) and raw mutation rates following gamma distribution realistic for nuclear protein coding genes? The dominant source of mutation rate variation in mammalian genes (used by the authors for testing the method) is likely to be the context-dependency, predominantly due to CpG contexts. Is the gamma distribution model capable of capturing this variation? Different rates for transitions between purines and pyrimidines imply strong strand bias. Is this a realistic assumption in nuclear genes and does it justify incorporation of additional parameters? Also, Tamura-Nei distance is known to have higher variance. How is this reflected in the performance of the method? I would suggest discussing these issues in the introduction and discussion sections.

#### Authors' response

*We are grateful to the valuable suggestions and revised relevant text accordingly. After it was brought forth by Professor Ziheng Yang in researching globin genes *[21], *gamma distribution has been widely used in characteristic of variable substitution in coding genes *[74], *especially in phylogeny analyses. In our method, we only computed the raw omega value (averaging all sites in a gene) based on raw mutation rates following gamma distribution. But this can be easily expanded to omega variations among sites in a manner using the sliding-window methods when necessary. It was proposed that nucleotide substitutions in both coding and noncoding regions are context-dependent in the sense that substitution rates depend on the identity of neighboring bases by adopting an approach of incorporating gamma distribution *[29]. *Furthermore, models that allow for the correlation of substitution rates at adjacent sites were also developed *[30]. *However, as these models tend to produce results similar to the simple gamma model and variations of α can make the distribution suitable for accommodating different levels of rate variation in various data sets *[31], *we chose the simple gamma distribution as the depiction of raw various mutation rates. As to the difference between purine and pyrimidine transitions, they are driven by potential underlying forces such as codon bias to the extent that it is correlated across codons in a gene and the chemistry (specificity) of DNA synthesis, damaging agents, DNA polymerase, and DNA repair enzymes *[73]. *In our computer simulations, we found that the new method did not always have higher variations in related parameter estimations as in compared with other methods*.

2) I suggest that the presentation of the manuscript will be improved. For example, it is not clear that by gamma distribution of substitution rates (and, in general, by variable substitution rates) the authors mean gamma distribution of raw mutation rate rather than gamma distribution of omega.

#### Authors' response

*Done. We revised the text to clarify this point. We do mean γ distribution of the raw mutation rate rather than omega*.

3) Tests on real data: I would suggest eliminating the discussion of positive selection between human and chimpanzee. Small number of substitutions and relaxation of selection due to small effective population size may easily lead to the observed increase in genes with Ka/Ks > 1. Also, I do not see why human-chimpanzee comparison would be a good test of the method because there are essentially no multiple hits, so any method including simple counting should be reliable. A good test would be the analysis of known examples of proteins evolving under positive or negative selection and demonstration that the new method has higher power to detect selection (e.g. using fewer species or partial sequences). I understand, however, that this may be a subject of a separate study.

#### Authors' response

*Agreed. We have removed the discussion on positive selection on the human-chimp dataset. We added an example for an evaluation of our new method. We are performing a systematic study on sequences from diverse evolutionary distances and planned to publish the results in separate manuscripts*.

4) Is the software implementation of the method available?

#### Authors' response

*Yes. Since the new integrated version of KaKs_Calculator 2.0 is still being programmed, a simple C++ source code package (can be used in Linux) is available upon request from the authors now*.

## Supplementary Material

Additional file 1 **Standard deviations of ω and supplementary method.** Additional file 1 contains: (I) the standard deviations of ω values that are used in Figure 1, Figure 2, Figure 4, Figure 5 and Table 4; (II) human codon and rice codon frequencies; and (III) an approach of determining the optimal α values in each test. Click here for file

Additional file 2 ****Description of the Tamura-Nei model and the detailed derivations of κ_R _and κ_Y_**.** Additional file 2 contains supplementary tables and derivation process in this study. This file contains two sections: section I shows the description of the Tamura-Nei model and section II details the procedures for deducing κ_R _and κ_Y_. Click here for file

## References

[B1] Gillespie JH (1991). The Causes of Molecular Evolution.

[B2] Kimura M (1983). The neutral theory of molecular evolution.

[B3] Li WH (1997). Molecular Evolution.

[B4] Yang Z (2006). Computational Molecular Evolution.

[B5] Hurst LD (2002). The Ka/Ks ratio: diagnosing the form of sequence evolution. Trends Genet.

[B6] Yang Z, Bielawski JP (2000). Statistical methods for detecting molecular adaptation. Trends Ecol Evol.

[B7] Nei M, Gojobori T (1986). Simple methods for estimating the numbers of synonymous and nonsynonymous nucleotide substitutions. Mol Biol Evol.

[B8] Li WH, Wu CI, Luo CC (1985). A new method for estimating synonymous and nonsynonymous rates of nucleotide substitution considering the relative likelihood of nucleotide and codon changes. Mol Biol Evol.

[B9] Li WH (1993). Unbiased estimation of the rates of synonymous and nonsynonymous substitution. J Mol Evol.

[B10] Pamilo P, Bianchi NO (1993). Evolution of the Zfx and Zfy genes: rates and interdependence between the genes. Mol Biol Evol.

[B11] Tzeng YH, Pan R, Li WH (2004). Comparison of three methods for estimating rates of synonymous and nonsynonymous nucleotide substitutions. Mol Biol Evol.

[B12] Zhang Z, Yu J (2006). Evaluation of six methods for estimating synonymous and nonsynonymous substitution rates. Genomics Proteomics Bioinformatics.

[B13] Yang Z, Nielsen R (2000). Estimating synonymous and nonsynonymous substitution rates under realistic evolutionary models. Mol Biol Evol.

[B14] Zhang Z, Li J, Yu J (2006). Computing Ka and Ks with a consideration of unequal transitional substitutions. BMC Evol Biol.

[B15] Fitch WM (1986). The estimate of total nucleotide substitutions from pairwise differences is biased. Philos Trans R Soc Lond B Biol Sci.

[B16] Fitch WM, Margoliash E (1967). A method for estimating the number of invariant amino acid coding positions in a gene using cytochrome c as a model case. Biochem Genet.

[B17] Fitch WM, Markowitz E (1970). An improved method for determining codon variability in a gene and its application to the rate of fixation of mutations in evolution. Biochem Genet.

[B18] Holmquist R, Goodman M, Conroy T, Czelusniak J (1983). The spatial distribution of fixed mutations within genes coding for proteins. J Mol Evol.

[B19] Uzzell T, Corbin KW (1971). Fitting discrete probability distributions to evolutionary events. Science.

[B20] Wakeley J (1993). Substitution rate variation among sites in hypervariable region 1 of human mitochondrial DNA. J Mol Evol.

[B21] Yang Z (1992). Variations of substitution rates and estimation of evolutionary distances of DNA sequence. PhD Thesis.

[B22] Jin L, Nei M (1990). Limitations of the evolutionary parsimony method of phylogenetic analysis. Mol Biol Evol.

[B23] Li WH, Gouy M, Sharp PM, O'HUigin C, Yang YW (1990). Molecular phylogeny of Rodentia, Lagomorpha, Primates, Artiodactyla, and Carnivora and molecular clocks. Proc Natl Acad Sci USA.

[B24] Tamura K, Nei M (1993). Estimation of the number of nucleotide substitutions in the control region of mitochondrial DNA in humans and chimpanzees. Mol Biol Evol.

[B25] Yang Z (1993). Maximum-likelihood estimation of phylogeny from DNA sequences when substitution rates differ over sites. Mol Biol Evol.

[B26] Yang Z (1994). Maximum likelihood phylogenetic estimation from DNA sequences with variable rates over sites: approximate methods. J Mol Evol.

[B27] Yang Z, Goldman N, Friday A (1994). Comparison of models for nucleotide substitution used in maximum-likelihood phylogenetic estimation. Mol Biol Evol.

[B28] Hasegawa M, Kishino H, Yano T (1985). Dating of the human-ape splitting by a molecular clock of mitochondrial DNA. J Mol Evol.

[B29] Siepel A, Haussler D (2004). Phylogenetic estimation of context-dependent substitution rates by maximum likelihood. Mol Biol Evol.

[B30] Felsenstein J, Churchill GA (1996). A Hidden Markov Model approach to variation among sites in rate of evolution. Mol Biol Evol.

[B31] Yang Z (1996). Among-site rate variation and its impact on phylogenetic analyses. Trends in Ecology & Evolution.

[B32] Comeron JM (1995). A method for estimating the numbers of synonymous and nonsynonymous substitutions per site. J Mol Evol.

[B33] Goldman N, Yang Z (1994). A codon-based model of nucleotide substitution for protein-coding DNA sequences. Mol Biol Evol.

[B34] Kimura M (1980). A simple method for estimating evolutionary rates of base substitutions through comparative studies of nucleotide sequences. J Mol Evol.

[B35] Muse SV, Gaut BS (1994). A likelihood approach for comparing synonymous and nonsynonymous nucleotide substitution rates, with application to the chloroplast genome. Mol Biol Evol.

[B36] Yang Z (1997). PAML: a program package for phylogenetic analysis by maximum likelihood. Comput Appl Biosci.

[B37] Hubbard T, Andrews D, Caccamo M, Cameron G, Chen Y, Clamp M, Clarke L, Coates G, Cox T, Cunningham F (2005). Ensembl 2005. Nucleic Acids Res.

[B38] Yu J, Wang J, Lin W, Li S, Li H, Zhou J, Ni P, Dong W, Hu S, Zeng C (2005). The Genomes of Oryza sativa: a history of duplications. PLoS Biol.

[B39] Jukes TH, Cantor CR (1969). Evolution of protein molecules. Mammalian Protein Metabolism.

[B40] Lio P, Goldman N (1998). Models of molecular evolution and phylogeny. Genome Res.

[B41] (2005). Initial sequence of the chimpanzee genome and comparison with the human genome. Nature.

[B42] Zhang Z, Li J, Zhao XQ, Wang J, Wong GK, Yu J (2006). KaKs_Calculator: calculating Ka and Ks through model selection and model averaging. Genomics Proteomics Bioinformatics.

[B43] Hirayasu K, Ohashi J, Tanaka H, Kashiwase K, Ogawa A, Takanashi M, Satake M, Jia GJ, Chimge NO, Sideltseva EW (2008). Evidence for natural selection on leukocyte immunoglobulin-like receptors for HLA class I in Northeast Asians. Am J Hum Genet.

[B44] Bakewell MA, Shi P, Zhang J (2007). More genes underwent positive selection in chimpanzee evolution than in human evolution. Proc Natl Acad Sci USA.

[B45] Nielsen R, Bustamante C, Clark AG, Glanowski S, Sackton TB, Hubisz MJ, Fledel-Alon A, Tanenbaum DM, Civello D, White TJ (2005). A scan for positively selected genes in the genomes of humans and chimpanzees. PLoS Biol.

[B46] Huelsenbeck JP, Dyer KA (2004). Bayesian estimation of positively selected sites. J Mol Evol.

[B47] Huelsenbeck JP, Jain S, Frost SW, Pond SL (2006). A Dirichlet process model for detecting positive selection in protein-coding DNA sequences. Proc Natl Acad Sci USA.

[B48] Pesole G, Saccone C (2001). A novel method for estimating substitution rate variation among sites in a large dataset of homologous DNA sequences. Genetics.

[B49] Pond SK, Muse SV (2005). Site-to-site variation of synonymous substitution rates. Mol Biol Evol.

[B50] Yang Z, Nielsen R (2002). Codon-substitution models for detecting molecular adaptation at individual sites along specific lineages. Mol Biol Evol.

[B51] Yang Z, Nielsen R, Goldman N, Pedersen AM (2000). Codon-substitution models for heterogeneous selection pressure at amino acid sites. Genetics.

[B52] Yang Z, Swanson WJ (2002). Codon-substitution models to detect adaptive evolution that account for heterogeneous selective pressures among site classes. Mol Biol Evol.

[B53] Anisimova M, Bielawski JP, Yang Z (2001). Accuracy and power of the likelihood ratio test in detecting adaptive molecular evolution. Mol Biol Evol.

[B54] Bielawski JP, Yang Z (2003). Maximum likelihood methods for detecting adaptive evolution after gene duplication. J Struct Funct Genomics.

[B55] Nielsen R, Yang Z (1998). Likelihood models for detecting positively selected amino acid sites and applications to the HIV-1 envelope gene. Genetics.

[B56] Yang Z (1998). Likelihood ratio tests for detecting positive selection and application to primate lysozyme evolution. Mol Biol Evol.

[B57] Yang Z (2007). PAML 4: phylogenetic analysis by maximum likelihood. Mol Biol Evol.

[B58] Pond SL, Frost SD, Muse SV (2005). HyPhy: hypothesis testing using phylogenies. Bioinformatics.

[B59] Clark AG, Glanowski S, Nielsen R, Thomas PD, Kejariwal A, Todd MA, Tanenbaum DM, Civello D, Lu F, Murphy B (2003). Inferring nonneutral evolution from human-chimp-mouse orthologous gene trios. Science.

[B60] Petersen L, Bollback JP, Dimmic M, Hubisz M, Nielsen R (2007). Genes under positive selection in Escherichia coli. Genome Res.

[B61] Richards S, Liu Y, Bettencourt BR, Hradecky P, Letovsky S, Nielsen R, Thornton K, Hubisz MJ, Chen R, Meisel RP (2005). Comparative genome sequencing of Drosophila pseudoobscura: chromosomal, gene, and cis-element evolution. Genome Res.

[B62] Cui P, Ji R, Ding F, Qi D, Gao H, Meng H, Yu J, Hu S, Zhang H (2007). A complete mitochondrial genome sequence of the wild two-humped camel (Camelus bactrianus ferus): an evolutionary history of camelidae. BMC Genomics.

[B63] Warren WC, Hillier LW, Marshall Graves JA, Birney E, Ponting CP, Grutzner F, Belov K, Miller W, Clarke L, Chinwalla AT (2008). Genome analysis of the platypus reveals unique signatures of evolution. Nature.

[B64] Zhu J, He F, Hu S, Yu J (2008). On the nature of human housekeeping genes. Trends Genet.

[B65] Benner SA, Trabesinger N, Schreiber D (1998). Post-genomic science: converting primary structure into physiological function. Adv Enzyme Regul.

[B66] Liberles DA (2001). Evaluation of methods for determination of a reconstructed history of gene sequence evolution. Mol Biol Evol.

[B67] Messier W, Stewart CB (1997). Episodic adaptive evolution of primate lysozymes. Nature.

[B68] Fares MA, Elena SF, Ortiz J, Moya A, Barrio E (2002). A sliding window-based method to detect selective constraints in protein-coding genes and its application to RNA viruses. J Mol Evol.

[B69] Siltberg J, Liberles DA (2002). A simple covarion-based approach to analyse nucleotide substitution rates. Journal of Evolutionary Biology.

[B70] Berglund AC, Wallner B, Elofsson A, Liberles DA (2005). Tertiary windowing to detect positive diversifying selection. J Mol Evol.

[B71] Suzuki Y (2004). Three-dimensional window analysis for detecting positive selection at structural regions of proteins. Mol Biol Evol.

[B72] NCBI HomoloGene. ftp://ftp.ncbi.nih.gov/pub/HomoloGene/.

[B73] Ota R, Penny D (2003). Estimating changes in mutational mechanisms of evolution. J Mol Evol.

[B74] Kumar S (1996). Patterns of nucleotide substitution in mitochondrial protein coding genes of vertebrates. Genetics.

