# Supplementary material for: γ-MYN: a new algorithm for estimating Ka and Ks with consideration of variable substitution rates
Source: Biol Direct. 2009 Jun 16;4:20. doi: 10.1186/1745-6150-4-20 (PMC2702329; doi:10.1186/1745-6150-4-20)
Supplement: Additional file 1 — Standard deviations of ω and supplementary method. Additional file 1 contains: (I) the standard deviations of ω values that are used in Figure 1, Figure 2, Figure 4, Figure 5 and Table 4; (II) human codon and rice codon frequencies; and (III) an approach of determining the optimal α values in each test. [file 1745-6150-4-20-S1.pdf]

Additional file 1

γ-MYN: a new algorithm for estimating Ka and Ks with consideration of variable substitution rates

Da-Peng Wang[*,1,2], Hao-Lei Wan[*,1,2] , Song Zhang[*,1,2,3], and Jun Yu[§1,3]

[1]CAS Key Laboratory of Genome Sciences and Information, Beijing Institute of Genomics, Chinese Academy of Sciences, Beijing 100029, China
[2]Graduate University of Chinese Academy of Sciences, Beijing 100039, China
[3]Institute of Computing Technology, Chinese Academy of Sciences, Beijing 100080, China

[*]These authors contributed equally to this work.
[§]Corresponding author

Table S2 – Standard deviations of ω in Figure 1(Estimated ω based on YN, MYN, and *γ*-MYN).

Codon Frequencies	Method κ_R \	ω=0.3 α=3.3			ω=1 α=20			ω=3 α=20		
		YN	MYN	γ-MYN	YN	MYN	γ-MYN	YN	MYN	γ-MYN
Equal Codon Frequencies	1	0.0753	0.0864	0.0866	0.2653	0.3007	0.3018	1.4545	1.6520	1.6587
	2	0.0770	0.0837	0.0839	0.2854	0.3081	0.3092	1.5206	1.6288	1.6352
	3	0.0785	0.0826	0.0829	0.3001	0.3136	0.3147	1.5197	1.5623	1.5682
	4	0.0785	0.0806	0.0808	0.3128	0.3166	0.3176	1.5756	1.5755	1.5815
	5	0.0806	0.0805	0.0807	0.3118	0.3099	0.3109	1.7287	1.7055	1.7115
	6	0.0805	0.0788	0.0790	0.3024	0.2954	0.2964	1.8146	1.7678	1.7741
	7	0.0801	0.0772	0.0775	0.3151	0.3006	0.3016	1.6249	1.5335	1.5394
	8	0.0828	0.0786	0.0788	0.3165	0.2985	0.2994	1.6900	1.5795	1.5855
	9	0.0853	0.0799	0.0801	0.3228	0.2986	0.2996	1.5386	1.4023	1.4075
	10	0.0850	0.0785	0.0788	0.3186	0.2910	0.2919	1.6125	1.4605	1.4658
Human Codon Frequencies	1	0.0706	0.0880	0.0885	0.2658	0.3134	0.3146	1.6489	1.9978	2.0051
	2	0.0743	0.0849	0.0855	0.2834	0.3162	0.3173	1.6793	1.7934	1.7993
	3	0.0761	0.0822	0.0826	0.3003	0.3168	0.3179	1.6326	1.6469	1.6524
	4	0.0777	0.0799	0.0803	0.3143	0.3171	0.3181	1.8438	1.8056	1.8124
	5	0.0798	0.0792	0.0796	0.3053	0.2964	0.2974	1.8528	1.7420	1.7475
	6	0.0825	0.0793	0.0796	0.3271	0.3091	0.3101	1.9010	1.7506	1.7560
	7	0.0852	0.0799	0.0802	0.3363	0.3107	0.3116	1.7862	1.6028	1.6081
	8	0.0867	0.0803	0.0807	0.3550	0.3234	0.3244	1.6793	1.4760	1.4811
	9	0.0886	0.0802	0.0806	0.3588	0.3215	0.3225	1.9581	1.6644	1.6702
	10	0.0900	0.0803	0.0808	0.3415	0.3005	0.3015	1.7863	1.5346	1.5399
Rice Codon Frequencies	1	0.0667	0.0834	0.0836	0.2578	0.3143	0.3155	1.4073	1.6538	1.6601
	2	0.0693	0.0800	0.0802	0.2841	0.3222	0.3233	1.5445	1.6856	1.6916
	3	0.0742	0.0808	0.0810	0.3080	0.3279	0.3290	1.8412	1.9363	1.9427
	4	0.0748	0.0783	0.0785	0.3030	0.3114	0.3124	1.6986	1.6968	1.7029
	5	0.0767	0.0769	0.0771	0.3195	0.3188	0.3198	1.8242	1.7644	1.7698
	6	0.0800	0.0778	0.0780	0.3097	0.2981	0.2991	2.0635	1.9281	1.9340
	7	0.0825	0.0779	0.0781	0.3231	0.3021	0.3030	1.7558	1.6116	1.6167
	8	0.0846	0.0773	0.0775	0.3388	0.3082	0.3091	2.2187	1.9844	1.9905
	9	0.0872	0.0783	0.0786	0.3471	0.3090	0.3100	2.0236	1.7541	1.7596
	10	0.0880	0.0776	0.0779	0.3916	0.3398	0.3407	2.2779	1.9314	1.9372

Table S3 - Standard deviations of ω in Figure 2 (Estimated ω when κ_Y = 3.75 and κ_R varies from 1 to 10 under negative selection).

ω/α	Method	κ_R									
		1	2	3	4	5	6	7	8	9	10
ω=0.1 α=4.8	YN	0.0287	0.0304	0.0315	0.0325	0.0335	0.0342	0.0356	0.0360	0.0365	0.0379
	MYN	0.0352	0.0346	0.0339	0.0333	0.0330	0.0326	0.0333	0.0331	0.0330	0.0336
	γ-MYN	0.0348	0.0343	0.0336	0.0329	0.0327	0.0323	0.0329	0.0327	0.0327	0.0333
ω=0.2 α=4	YN	0.0501	0.0491	0.0513	0.0543	0.0566	0.0583	0.0587	0.0602	0.0620	0.0639
	MYN	0.0625	0.0564	0.0554	0.0560	0.0565	0.0561	0.0552	0.0559	0.0562	0.0570
	γ-MYN	0.0624	0.0563	0.0553	0.0558	0.0564	0.0560	0.0550	0.0557	0.0561	0.0568
ω=0.3 α=3.3	YN	0.0706	0.0743	0.0761	0.0777	0.0798	0.0825	0.0852	0.0867	0.0886	0.0900
	MYN	0.0880	0.0849	0.0822	0.0799	0.0792	0.0793	0.0799	0.0803	0.0802	0.0803
	γ-MYN	0.0885	0.0855	0.0826	0.0803	0.0796	0.0796	0.0802	0.0807	0.0806	0.0808
ω=0.4 α=3	YN	0.0937	0.0970	0.1005	0.1069	0.1083	0.1125	0.1159	0.1197	0.1228	0.1218
	MYN	0.1149	0.1100	0.1083	0.1103	0.1074	0.1081	0.1078	0.1102	0.1099	0.1082
	γ-MYN	0.1163	0.1113	0.1096	0.1116	0.1085	0.1092	0.1089	0.1112	0.1109	0.1092
ω=0.5 α=2.5	YN	0.1166	0.1223	0.1278	0.1339	0.1419	0.1464	0.1443	0.1471	0.1518	0.1541
	MYN	0.1430	0.1387	0.1377	0.1374	0.1396	0.1402	0.1345	0.1342	0.1365	0.1359
	γ-MYN	0.1459	0.1413	0.1401	0.1399	0.1419	0.1424	0.1365	0.1363	0.1387	0.1379
ω=0.6 α=2	YN	0.1496	0.1522	0.1585	0.1635	0.1724	0.1767	0.1825	0.1814	0.1828	0.1869
	MYN	0.1817	0.1716	0.1684	0.1661	0.1702	0.1689	0.1702	0.1651	0.1626	0.1648
	γ-MYN	0.1873	0.1763	0.1727	0.1703	0.1744	0.1730	0.1743	0.1691	0.1665	0.1688
ω=0.7 α=1.5	YN	0.1754	0.1769	0.1916	0.1996	0.2071	0.2112	0.2184	0.2161	0.2161	0.2153
	MYN	0.2108	0.1983	0.2035	0.2012	0.2022	0.2015	0.2045	0.1967	0.1926	0.1881
	γ-MYN	0.2204	0.2063	0.2114	0.2087	0.2097	0.2090	0.2121	0.2040	0.1997	0.1950
ω=0.8 α=1.5	YN	0.2002	0.2143	0.2303	0.2390	0.2455	0.2452	0.2519	0.2607	0.2536	0.2621
	MYN	0.2389	0.2389	0.2434	0.2408	0.2390	0.2327	0.2354	0.2371	0.2263	0.2286
	γ-MYN	0.2502	0.2492	0.2535	0.2503	0.2483	0.2419	0.2446	0.2463	0.2353	0.2376
ω=0.9 α=1.5	YN	0.2325	0.2500	0.2605	0.2733	0.2739	0.2797	0.2911	0.3056	0.3080	0.2967
	MYN	0.2755	0.2793	0.2776	0.2764	0.2662	0.2661	0.2698	0.2784	0.2748	0.2594
	γ-MYN	0.2894	0.2923	0.2898	0.2880	0.2776	0.2775	0.2809	0.2898	0.2860	0.2701

Table S4 - Standard deviations of ω in Figure 4(The effect of *t* based on YN, MYN, and *γ*-MYN).

ω	Method	*t*									
		0.1	0.2	0.3	0.4	0.5	0.6	0.7	0.8	0.9	1
ω=0.2	YN	0.2022	0.1349	0.1021	0.0915	0.0828	0.0770	0.0764	0.0746	0.0732	0.0727
	MYN	0.1719	0.1084	0.0816	0.0725	0.0648	0.0605	0.0585	0.0573	0.0566	0.0559
	γ-MYN	0.1718	0.1082	0.0814	0.0721	0.0644	0.0601	0.0581	0.0567	0.0561	0.0555
ω=0.3	YN	0.3318	0.1995	0.1669	0.1330	0.1149	0.1129	0.1078	0.1053	0.1022	0.1001
	MYN	0.2792	0.1597	0.1324	0.1054	0.0903	0.0880	0.0844	0.0827	0.0800	0.0787
	γ-MYN	0.2795	0.1599	0.1327	0.1055	0.0904	0.0880	0.0845	0.0829	0.0803	0.0793
ω=0.4	YN	0.4404	0.2767	0.3006	0.1788	0.1570	0.1453	0.1439	0.1343	0.1327	0.1270
	MYN	0.3685	0.2245	0.2520	0.1426	0.1246	0.1151	0.1128	0.1065	0.1050	0.0998
	γ-MYN	0.3693	0.2252	0.2533	0.1432	0.1253	0.1158	0.1138	0.1076	0.1064	0.1012

Table S5 - Standard deviations of ω in Figure 5(The effects of κ_R and κ_Y based on YN, MYN, and *γ*-MYN).

ω	Method	$\kappa_R = \kappa_Y$									
		1	2	3	4	5	6	7	8	9	10
ω=0.2	YN	0.0561	0.0541	0.0529	0.0536	0.0544	0.0539	0.0544	0.0540	0.0543	0.0539
	MYN	0.0579	0.0560	0.0551	0.0560	0.0571	0.0569	0.0575	0.0572	0.0574	0.0571
	γ-MYN	0.0581	0.0561	0.0550	0.0559	0.0569	0.0567	0.0572	0.0569	0.0571	0.0567
ω=0.3	YN	0.0826	0.0789	0.0776	0.0760	0.0752	0.0756	0.0766	0.0757	0.0762	0.0763
	MYN	0.0852	0.0813	0.0804	0.0791	0.0785	0.0790	0.0806	0.0793	0.0797	0.0800
	γ-MYN	0.0857	0.0817	0.0808	0.0793	0.0788	0.0792	0.0807	0.0794	0.0798	0.0801

Table S6 - Standard deviations of S%, Ka, Ks and ω in Table 4(Proportions of synonymous sites (S%) and estimates of Ka , Ks and ω).

Method	$\kappa_R - \kappa_Y > 0.4$				$\kappa_R - \kappa_Y < -0.4$				$\lvert\kappa_R - \kappa_Y\rvert \le 0.4$			
	S%	Ka	Ks	ω	S%	Ka	Ks	ω	S%	Ka	Ks	ω
human-dog orthologs												
GY	0.000011	0.000035	0.002674	0.001886	0.000360	0.000093	0.000486	0.000333	0.000354	0.000147	0.001448	0.000191
YN	0.000354	0.000004	0.002939	0.002824	0.000411	0.000105	0.000678	0.000335	0.000530	0.000189	0.001811	0.000349
MYN	0.000440	0.000010	0.003549	0.002627	0.000455	0.000102	0.000593	0.000335	0.000571	0.000193	0.002005	0.000406
γ-MYN	0.000441	0.000003	0.004083	0.002572	0.000462	0.000094	0.000746	0.000303	0.000568	0.000204	0.002339	0.000397
human-mouse orthologs												
GY	0.000036	0.000702	0.006937	0.000133	0.000409	0.001202	0.005636	0.000278	0.000534	0.000843	0.021609	0.001204
YN	0.000026	0.000665	0.001338	0.000499	0.000487	0.000971	0.005337	0.000087	0.000665	0.000814	0.009187	0.001210
MYN	0.000023	0.000648	0.001245	0.000429	0.000563	0.000965	0.004935	0.000100	0.000698	0.000813	0.001936	0.001158
γ-MYN	0.000020	0.000667	0.000265	0.000560	0.000567	0.000995	0.004167	0.000237	0.000709	0.000852	0.001028	0.001057
human-chimp orthologs												
GY	0.000720	0.000064	0.000528	0.019020	0.000566	0.000118	0.000311	0.000076	0.000552	0.000007	0.000030	0.001191
YN	0.000899	0.000089	0.000517	0.020643	0.000672	0.000151	0.000302	0.000113	0.000225	0.000276	0.000124	0.000420
MYN	0.000961	0.000088	0.000581	0.020185	0.000708	0.000156	0.000269	0.000157	0.000234	0.000262	0.000126	0.000459
γ-MYN	0.000961	0.000093	0.000600	0.020277	0.000708	0.000171	0.000281	0.000168	0.000234	0.000281	0.000144	0.000453

Table S7 – Human codon frequencies and rice codon frequencies are listed.

A.

human			
0.017409269	0.020289522	0.007486065	0.012938188
0.014594883	0.017283519	0.011678701	0.004546929
0.012389283	0.01538331	0	0
0.01040624	0.012300461	0	0.012483096
0.013075914	0.01937834	0.009098848	0.040017166
0.017203679	0.019580936	0.016632818	0.006966103
0.010930194	0.015058957	0.012153753	0.034648875
0.004700622	0.010643766	0.006286459	0.011740578
0.016102875	0.020771561	0.007198639	0.022269572
0.012899266	0.018333425	0.014548975	0.00597508
0.017131822	0.019294507	0.025269587	0.033415336
0.011979102	0.019483131	0.011901257	0.01160884
0.011028997	0.014318435	0.007056921	0.028037066
0.018568955	0.028328485	0.015867345	0.007531974
0.02230051	0.025542044	0.029791566	0.040795613
0.0107266	0.022306499	0.016356369	0.015953173

B.

rice			
0.0133796	0.0235996	0.00596498	0.0144556
0.0135125	0.0173109	0.0138465	0.0124041
0.010018	0.0161527	0	0
0.0060901	0.0125415	0	0.0129919
0.0160226	0.0271046	0.0070549	0.0227605
0.0136438	0.0117307	0.0143695	0.016982
0.0113064	0.0133664	0.0125183	0.0218757
0.0063044	0.0151675	0.0050089	0.0115529
0.0146539	0.0198529	0.0089378	0.0249317
0.0107111	0.0145815	0.0118241	0.0105799
0.0156193	0.018845	0.015647	0.0335305
0.0091412	0.0166212	0.0100826	0.0156274
0.0164041	0.0202853	0.0068767	0.0248971
0.0203679	0.0302987	0.01801	0.0252536
0.0257348	0.0280494	0.0209591	0.0381575
0.0150104	0.0286005	0.0148437	0.0160269

Note: 64 codon frequencies are in fixed order TTT, TTC, TTA, TTG, TCT, TCC, ..., GGG, under the F3x4 table.

Supplementary methods

A method of determining the optimal α values in each test

To select the optimal parameter "α", we build an **optimal index**.

$$f = \sum_{1 \le i \le 10} (C_i^{estimated} - C_i^{\mathrm{exp}ected})^2$$

In this function, the values of *i* from 1 to 10 stand for the κ_R values increasing from 1 to 10, fixing κ_Y =3.75, as used in the analyses. $C_i^{estimated}$ stands for the estimated values of *ω,* and $C_i^{\mathrm{exp}ected}$ stands for the expected values of *ω, when* κ_R = *i*. This function weighs the deviation from true values, regardless if the deviation is positive or negative. To minimize *f*, we get the optimal *a* under various conditions to force the estimated values to deviate from real values.
