# Supplementary material for: γ-MYN: a new algorithm for estimating Ka and Ks with consideration of variable substitution rates
Source: Biol Direct. 2009 Jun 16;4:20. doi: 10.1186/1745-6150-4-20 (PMC2702329; doi:10.1186/1745-6150-4-20)
Supplement: Additional file 2 — Description of the Tamura-Nei model and the detailed derivations of κR and κY. Additional file 2 contains supplementary tables and derivation process in this study. This file contains two sections: section I shows the description of the Tamura-Nei model and section II details the procedures for deducing κR and κY. [file 1745-6150-4-20-S2.pdf]

Additional file 2

γ-MYN: a new algorithm for estimating Ka and Ks with consideration of variable substitution rates

Da-Peng Wang[*,1,2], Hao-Lei Wan[*,1,2] , Song Zhang[*,1,2,3], and Jun Yu[§1,3]

[1]CAS Key Laboratory of Genome Sciences and Information, Beijing Institute of Genomics, Chinese Academy of Sciences, Beijing 100029, China
[2]Graduate University of Chinese Academy of Sciences, Beijing 100039, China
[3]Institute of Computing Technology, Chinese Academy of Sciences, Beijing 100080, China

[*]These authors contributed equally to this work.
[§]Corresponding author

1. Tamura-Nei Model

Table S1 Nucleotide Substitution Models

original nucleotide	mutant nucleotide			
	T	C	A	G
T	–	$\alpha_2 g_C$	βg_A	βg_G
C	$\alpha_2 g_T$	–	βg_A	βg_G
A	βg_T	βg_C	–	$\alpha_1 g_G$
G	βg_T	βg_C	$\alpha_1 g_A$	–

Note: α_1 ,transitional rate between purines; α_2 ,transitional rate between pyrimidines; β , transversional rate; g_N , frequencies of nucleotide N, where $N \in \{T, C, A, G\}$.

Under the assumption that the rate of nucleotide substitution λ is the same for all sites considered, Tamura and Nei used g_T , g_C , g_A and g_G to represent nucleotide

frequencies for T,C,A and G, respectively. They defined α_1, α_2 and β as transitional rates between purines and between pyrimidines, and transversional rate, respectively. They derived the formulas (S1-S3) for the proportions of transitional differences between purines(P_1) and between pyrimidines(P_2) and of transversional differences(Q) over divergence time t [1, 2]:

$$P_1 = \frac{2g_A g_G}{g_R}\{g_R + g_Y \exp(-2\beta t) - \exp[-2(g_R\alpha_1 + g_Y\beta)t]\} \quad \text{(S1)}$$

$$P_2 = \frac{2g_T g_C}{g_Y}\{g_Y + g_R \exp(-2\beta t) - \exp[-2(g_Y\alpha_2 + g_R\beta)t]\} \quad \text{(S2)}$$

$$Q = 2g_R g_Y[1 - \exp(-2\beta t)] \quad \text{(S3)}$$

where $g_R = g_A + g_G$ and $g_Y = g_T + g_C$.

2. Derivation of κ_R and κ_Y

Under the assumption that the rate of nucleotide substitution λ approximately follows the gamma distribution, we derive the equations for estimating κ_R and κ_Y.

We consider Tamura-Nei Model, where the average substitution rate is given by [1] $\lambda = 2g_A g_G\alpha_1 + 2g_T g_C\alpha_2 + 2g_R g_Y\beta$, where $g_R = g_A + g_G$ and $g_Y = g_T + g_C$. We assume that λ varies with nucleotide site according to the following gamma distribution [3, 4] :

$$f(\lambda) = \frac{b^\alpha}{\tau(\alpha)} e^{-b\lambda}\lambda^{\alpha-1} \quad \text{(S4)}$$

Where $\alpha = \bar{\lambda}^2 / V(\lambda)$ and $b = \alpha / \bar{\lambda}$, $\bar{\lambda}$ and $V(\lambda)$ being, respectively, the mean and variance of λ. $\tau(\alpha)$ is the gamma function. Here note that α is the square of the inverse of the coefficient of variation. To avoid using too many parameters, we set *b* = α so that the mean of the distribution is 1, with variance $1/\alpha$. The shape parameter α is then inversely related to the extent of rate variation at sites.

Therefore, if λ or α_1, α_2 and β follow the gamma distribution, the means of P_1, P_2 and Q are given by [1, 3, 4]

$$\overline{P_1} = \int_0^\infty P_1 f(\lambda) d\lambda = \frac{2g_A g_G}{g_R} \left\{ g_R - \left[\frac{\alpha}{\alpha + 2(g_R \overline{\alpha_1} + g_Y \overline{\beta})t} \right]^\alpha + g_Y \left(\frac{\alpha}{\alpha + 2\overline{\beta}t} \right)^\alpha \right\}, \text{(S5)}$$

$$\overline{P_2} = \int_0^\infty P_2 f(\lambda) d\lambda = \frac{2g_T g_C}{g_Y} \left\{ g_Y - \left[\frac{\alpha}{\alpha + 2(g_Y \overline{\alpha_2} + g_R \overline{\beta})t} \right]^\alpha + g_R \left(\frac{\alpha}{\alpha + 2\overline{\beta}t} \right)^\alpha \right\}, \text{(S6)}$$

$$\overline{Q} = \int_0^\infty Q f(\lambda) d\lambda = 2g_R g_Y \left[1 - \left(\frac{\alpha}{\alpha + 2\overline{\beta}t} \right)^\alpha \right] \quad \text{(S7)}$$

Where $\overline{\alpha_1}$, $\overline{\alpha_2}$ and $\overline{\beta}$ are the means of α_1, α_2 and β, respectively.

From (S5), (S6) and (S7) we can get the transformation:

$$2\overline{\alpha_1}t = \frac{\alpha}{g_R}[(1 - \frac{1}{2g_R}\overline{Q} - \frac{g_R}{2g_A g_G}\overline{P_1})^{-1/\alpha} - g_Y(1 - \frac{1}{2g_R g_Y}\overline{Q})^{-1/\alpha} - g_R], \quad \text{(S8)}$$

$$2\overline{\alpha_2}t = \frac{\alpha}{g_Y}[(1 - \frac{1}{2g_Y}\overline{Q} - \frac{g_Y}{2g_T g_C}\overline{P_2})^{-1/\alpha} - g_R(1 - \frac{1}{2g_R g_Y}\overline{Q})^{-1/\alpha} - g_Y], \quad \text{(S9)}$$

$$2\overline{\beta}t = \alpha[(1 - \frac{1}{2g_R g_Y}\overline{Q})^{-1/\alpha} - 1] \quad \text{(S10)}$$

In order to conveniently remember the meanings of $\overline{P_1}$, $\overline{P_2}$ and $\overline{Q}$, in our main manuscript we rename them as T_R, T_Y ,V, respectively. Hence, the formulas for estimating κ_R, κ_Y and *d* are as follows.

$$\kappa_R = \overline{\alpha_1} / \overline{\beta} = \frac{h - g_Y \times j - g_R}{g_R \times j - g_R}, \quad \text{(S11)}$$

$$\kappa_Y = \overline{\alpha_2} / \overline{\beta} = \frac{i - g_R \times j - g_Y}{g_Y \times j - g_Y} \quad \text{(S12)}$$

$$\begin{aligned} d &= 4g_A g_G \overline{\alpha_1}t + 4g_T g_C \overline{\alpha_2}t + 4g_R g_Y \overline{\beta}t \\ &= 2\alpha \left[\frac{g_A g_G}{g_R} h + \frac{g_T g_C}{g_Y} i + \left(g_R g_Y - \frac{g_A g_G g_Y}{g_R} - \frac{g_T g_C g_R}{g_Y} \right) j - g_A g_G - g_T g_C - g_R g_Y \right] \end{aligned}$$

(s13)

Where

$$h = \left(1 - \frac{1}{2g_R} V - \frac{g_R}{2g_A g_G} T_R\right)^{-1/\alpha}, \quad \text{(S14)}$$

$$i = \left(1 - \frac{1}{2g_Y} V - \frac{g_Y}{2g_T g_C} T_Y\right)^{-1/\alpha}, \quad \text{(S15)}$$

$$j = \left(1 - \frac{1}{2g_R g_Y} V\right)^{-1/\alpha} \quad \text{(S16)}$$

Reference

1. Tamura K, Nei M: **Estimation of the number of nucleotide substitutions in the control region of mitochondrial DNA in humans and chimpanzees.** *Mol Biol Evol* 1993, **10:**512-526.
2. Zhang Z, Li J, Yu J: **Computing Ka and Ks with a consideration of unequal transitional substitutions.** *BMC Evol Biol* 2006, **6:**44.
3. Jin L, Nei M: **Limitations of the evolutionary parsimony method of phylogenetic analysis.** *Mol Biol Evol* 1990, **7:**82-102.
4. Nei M, Gojobori T: **Simple methods for estimating the numbers of synonymous and nonsynonymous nucleotide substitutions.** *Mol Biol Evol* 1986, **3:**418-426.
